# Physical activity for children with chronic disease; a narrative review and practical applications

**DOI:** 10.1186/s12887-018-1377-3

**Published:** 2019-01-08

**Authors:** Sarah L. West, Laura Banks, Jane E. Schneiderman, Jessica E. Caterini, Samantha Stephens, Gillian White, Shilpa Dogra, Greg D. Wells

**Affiliations:** 1 Department of Biology, Trent/Fleming School of Nursing, Trent University, Toronto, Canada; 2 Translational Medicine, The Hospital for Sick Children, Toronto, Canada; 3 University Health Network, Toronto, Canada; 4 Faculty of Kinesiology and Physical Education, The University of Toronto, Toronto, Canada; 5 Child Health Evaluative Sciences, The Hospital for Sick Children, Toronto, Canada; 6 Institute of Health Policy Management and Evaluation, The University of Toronto, Toronto, Canada; 7 Faculty of Health Sciences (Kinesiology), University of Ontario Institute of Technology, Oshawa, Canada; 8 Translational Medicine, The Hospital for Sick Children, Peter Gilgan Centre for Research and Learning, 10th floor, 686 Bay St., Toronto, ON M5G 0A4 Canada

**Keywords:** Exercise, Medicine, Pediatric, Physical activity, Chronic disease, Children

## Abstract

**Background:**

Physical activity (PA) is associated with a diverse range of health benefits. International guidelines suggest that children should be participating in a minimum of 60 min of moderate to vigorous intensity PA per day to achieve these benefits. However, current guidelines are intended for healthy children, and thus may not be applicable to children with a chronic disease. Specifically, the dose of PA and disease specific exercise considerations are not included in these guidelines, leaving such children with few, if any, evidence-based informed suggestions pertaining to PA. Thus, the purpose of this narrative review was to consider current literature in the area of exercise as medicine and provide practical applications for exercise in five prevalent pediatric chronic diseases: respiratory, congenital heart, metabolic, systemic inflammatory/autoimmune, and cancer.

**Methods:**

For each disease, we present the pathophysiology of exercise intolerance, summarize the pediatric exercise intervention research, and provide PA suggestions.

**Results:**

Overall, exercise intolerance is prevalent in pediatric chronic disease. PA is important and safe for most children with a chronic disease, however exercise prescription should involve the entire health care team to create an individualized program.

**Conclusions:**

Future research, including a systematic review to create evidence-based guidelines, is needed to better understand the safety and efficacy of exercise among children with chronic disease.

## Background

Physical activity (PA), that is, any bodily movement produced by skeletal muscles that requires energy expenditure [1], is associated with many physiological and psychological health benefits across the lifespan. As such, multiple agencies including The Canadian Society for Exercise Physiology (CSEP), The American College of Sports Medicine (ACSM) and The World Health Organization (WHO) have published PA guidelines targeting all age groups including children, adults, and older adults [2–5]. For children aged 5–17 years, guidelines consistently recommend participating in at least 60 min of daily moderate–to-vigorous intensity PA (MVPA) daily [2], and clearly indicate that a greater volume of PA is associated with greater health benefit [6]. In fact, the emerging concept of using exercise as medicine implies that exercise can be used in a dose-dependent manner (akin to pharmaceutical drugs) to positively impact health outcomes for individuals with chronic diseases [7].

**Fig. 1:**
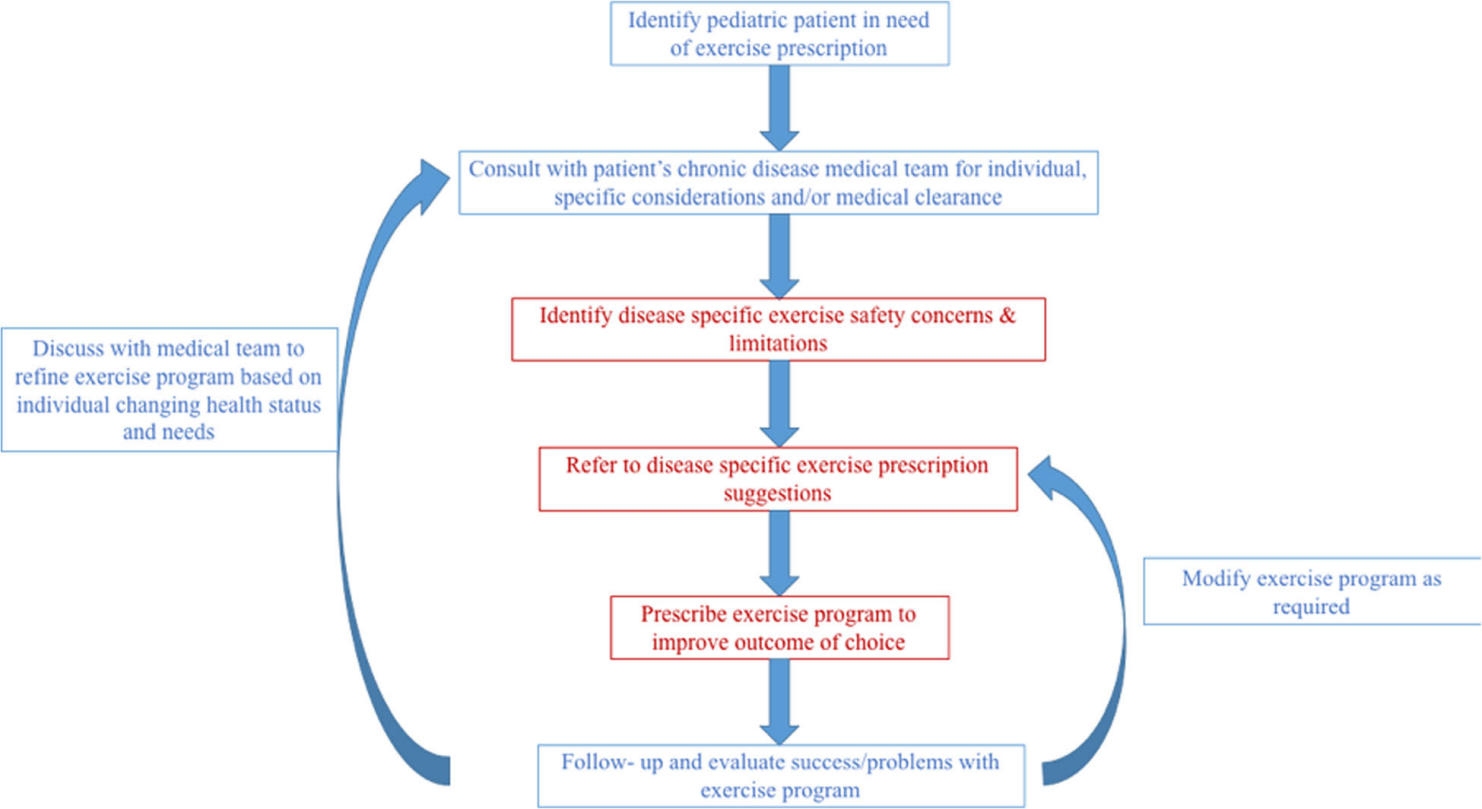
Flow chart of the use exercise as medicine and current suggestions for pediatric chronic disease. Legend: Red text identifies steps in the process that the current narrative reivew may help inform

Both PA & exercise (i.e., purposeful and intentional PA) may impact chronic disease by preventing the development of new chronic diseases (such as in metabolic syndrome), by directly modifying the disease (disease reversal, such as in type 2 diabetes) and/or by helping to manage the symptoms associated with chronic disease (such as in arthritis or cancer) [8]. As such, clinical practice guidelines for a variety of chronic diseases include guidance on the dose of PA that should be prescribed to individuals affected by that disease. However, these clinical practice guidelines often focus on adults, while guidelines that address chronic diseases that primarily affect children often ignore PA. Although the published international PA guidelines indicate how much exercise is suggested for otherwise healthy children [2–4], pediatric disease-specific PA guidelines are lacking. As a result, clinicians remain unsure about the dose of PA or appropriate modes of PA to prescribe to their patients. While healthy children should strive to achieve the recommended PA guidelines of 60 min of daily MVPA, the optimal prescription for children with a chronic disease requires greater specificity as well as careful consideration of risks and benefits (Fig. 1). Similar to pharmacotherapy, the dose of PA (i.e., the frequency, type, intensity, and time) may change depending on the chronic disease, and the child’s health and fitness levels.

Therefore, the purpose of this narrative review was to consider current literature in the area of exercise as medicine in prevalent pediatric chronic diseases, and provide practical applications for exercise prescription. We examined five common pediatric chronic diseases [9]: 1) respiratory, 2) congenital heart, 3) metabolic, 4) systemic inflammatory/autoimmune, and 5) cancer. We discuss the pathophysiology of exercise intolerance, summarize exercise intervention research, and provide practical applications for exercise in each pediatric cohort (Table 1).

**Table 1:** Summary of practical applications for the use of exercise as medicine for pediatric chronic disease. Note that these suggestions are **not** formal exercise recommendations; rather suggesions based on the current narrative review

Disease	Aerobic Exercise	HIIT Exercise	Resistance Training	Flexibility Training	General Comments
CF	F: Min 2x/week, progressive up to 7x/week I: Moderate intensity (~ 70% max HR); progressive T: 30–45 min sessions; progressive Ty: Aerobic activities (e.g. Running, swimming, cycling)	F: 2x/week I: > 75% max HR; progressive T: 30 min sessions; progressive, ensure adequate rest between work sessions (> 2 min); Ty: Interval training	F: 2-3x/week, non-consecutive days I: Moderate intensity, 70% of peak workload; progressive T: 3–5 sets of 10 repetitions; progressive Ty: body-weight exercises; supervised use of weights	F: 2x/week I: Not determined T: 15–20 min Ty: Yoga, stretching	Overall goal for exercise prescription in most chronic diseases is to have the patient achieve PA guidelines of 60 min/day using a combination of types of exercises described. Refer to disease sections for discussion of special considerations during exercise.
Asthma	F: Min 3x/week, progressive up to 7x/week I: Moderate intensity (~ 70% max HR); progressive T: Progressive to 60 min/session Ty: Aerobic activities (e.g. indoor soccer, cycling)	No evidence to support safety. Avoid at this time	F: 2-3x/week, non-consecutive days I: Moderate intensity, 70% of peak workload; progressive T: 1–2 sets of 8–15 repetitions; progressive to more Ty: body-weight exercises; supervised use of weights	F: 2x/week I: Not determined T: 15–20 min Ty: Yoga, stretching	
CHD	F: Progressive up to 7x/week I: Mild to moderate/high intensity (40–85% max HR); progressive T: Progressive to 60 min/session Ty: Aerobic activities (e.g., running, cycling, dance)	No evidence to support safety. Avoid at this time	F: 2-3x/week, non-consecutive days I: Low to moderate intensity (40–70% of peak workload); progressive T: 1–2 sets of 8–15 repetitions Ty: body-weight exercises; low resistance; supervised use of weights	F: 2x/week I: Not determined T: 15–20 min Ty: Yoga, stretching	
Obesity & T2D	F: Progressive up to 7x/week I: Moderate to high intensity (70–85% max HR); progressive T: Progressive to 60 min/session Ty: Aerobic activities (e.g., running, cycling, swimming)	F: 2 x/week I: 70–85% max HR; progressive T: 30 min sessions; ensure adequate rest between work sessions (> 2 min); progressive Ty: Interval training Note: No evidence to support safety in T2D, avoid at this time	F: 2-3x/week, non-consecutive days I: Moderate intensity (70% of peak workload); progressive T: 1–3 sets of 8–15 repetitions; increase resistance progressively Ty: body-weight exercises; supervised use of weights	F: 2x/week I: Not determined T: 15–20 min Ty: Yoga, stretching	
JIA	F: 2–3 x/week I: Moderate to high intensity (65–85% max HR); progressive T: 45–60 min/session; progressive Ty: Aerobic activities (e.g., running, cycling, swimming)	No evidence to support safety. Avoid at this time	F: 2-3x/week, non-consecutive days I: Low to moderate intensity (40–70% peak workload); progressive T: 1–3 sets of 8–15 repetitions; increase resistance progressively Ty: body-weight exercises; use of resistance bands; supervised use of weights	F: 2x/week I: Not determined T: 15–20 min Ty: Yoga, stretching	
Cancer	F: 2–4 x/week; progressive up to 3–5 x/week I: Start low, mild intensity (40% max HR); progressive up to moderate intensity (70% max HR) T: 30–45 min/session; progressive (can break into shorter 10 min blocks) Ty: Aerobic activities (e.g., running, cycling, swimming)	No evidence to support safety. Avoid at this time	F: 1-3x/week, non-consecutive days I: Low-moderate intensity (40–60% of peak workload); progressive T: 1–3 sets of 8–15 repetitions; increase resistance progressively Ty: body-weight exercises; use of resistance bands; supervised use of weights	F: 2x/week I: Not determined T: 15–20 min Ty: Yoga, stretching, balancing	

*CF* Cystic Fibrosis

*CHD* Congenital Heart Disease

*T2D* Type 2 Diabetes

*JIA* Juvenile Idiopathic Arthritis

*F* Frequency of exercise

*I* Intensity of exercise

*T* Time, i.e., amount of exercise

*Ty* Type of exercise

*HR* Heart rate

## Respiratory disease

i. Cystic Fibrosis

- *Pathophysiology of Exercise Intolerance in Pediatric Patients with Cystic Fibrosis*

Cystic Fibrosis (CF) is an autosomal-dominant disease occurring in approximately 1:2500–4000 live births per year in Canada and the United States [10, 11], with a higher incidence in European countries [12]. CF involves abnormal expression or function of the CF transmembrane conductance regulator protein, which results in thicker mucus production and associated complications to multiple organ systems (especially digestive and respiratory) [13, 14]. Exercise capacity is reduced in pediatric CF due to multiple factors, including lung and cardiovascular function [15], peripheral skeletal muscle function [16], and poor nutritional status [15, 17, 18].

Individuals with CF increase their ventilation during exercise to adapt to the increased dead space in their lungs [19], and increased work of breathing diverts blood flow from the exercise muscles. Oxygen desaturation occurs in moderate-to-severe CF due to ventilation/perfusion mismatching. Investigators have found that ventilatory limitations to exercise mostly exist in patients with severe CF (the amount of air you can exhale from your lungs in one second [FEV_1_] < 40% predicted), and that respiratory factors are not the primary exercise limitation in mild to moderate CF [20, 21]. Cardiovascular complications in CF are poorly described, but there is evidence for abnormalities in right ventricular systolic function [22] and diastolic function [23]. Both large artery [24] and endothelial microvascular dysfunction have been reported in CF, and may affect the peripheral skeletal muscles’ ability to direct blood flow to areas of increased demand during exercise. Indeed, impaired endothelial function is correlated with both workload and ventilation in CF patients at peak exercise [25].

Children with CF also experience a nonspecific impact of systemic disease on skeletal muscle function [26]. Notably, patients exhibit a lower resting adenosine riphosphate (ATP)/phosphocreatine (PCr) ratio and slower PCr recovery time values compared with healthy controls [26], which may result in a mismatch between the exercise demands and the metabolic capacity of skeletal muscle. Individuals with CF also have muscle atrophy, which can be due to decreased nutritional status and increased basal levels of inflammatory cytokines [27, 28]. Overall, there are many factors that contribute to poor PA and exercise tolerance in children with CF.

b. *Exercise in Pediatric Patients with* CF

Significant benefits of exercise and habitual PA have been documented for children with CF [29, 30] including improvements in cardiovascular endurance [31, 32], muscular strength [30, 33], quality of life [34, 35], and mucus clearance [36, 37].

There are few randomized controlled exercise intervention trials (EX-RCT) in pediatric patients with CF. Of these, one evaluated the difference between aerobic (70% peak heart rate for 30 min) versus resistance training (70% of peak workload, 5 sets of 10 repetitions) following hospital admission, and observed improved FEV_1_ and maximal aerobic capacity (VO_2peak_) following discharge [33]. Other hospital-based EX-RCTs include a 12-week treadmill training session, twice a week for 30 min at 60% of the peak heart rate achieved during exercise testing, and increases in VO_2peak_ but no changes in FEV_1_ were observed [32]. A supervised 8 week combination of resistance training, cycle erogmetery, and active play three times per week improved VO_2peak_ [38], and when in combination with two days per week of inspiratory muscle training, improvements in inspiratory pressure were also observed relative to controls [39]. Finally, a three-year home exercise program of 20 min aerobic exercise, 3 days per week, resulted in a slower decline in percent predicted forced vital capacity and forced expiratory volume in 1 min [40].

Anaerobic exercise (including high intensity interval training; HIIT) also improves both anaerobic performance and health-related quality of life in children with CF [14, 34, 35]. One study found that anaerobic training (20–30 s bouts at maximal speed) for 30–45 min a day, two days a week for 12 weeks increased both peak power and VO_2peak_ in children with CF, and the anaerobic benefits of increased peak power were sustained at 12-week follow-up [34].

c. *Practical Applications for the use of Exercise as Medicine in Pediatric Patients with* CF

Prior to engaging in a new exercise program, children with CF should undergo exercise testing to identify maximal heart rate, levels at which oxygen desaturation and ventilation limits occur, exercise-related bronchospasm, and response to therapy so that the safest exercise program can be designed [41]. A recent position statement suggests that exercise testing (such as The Godfrey Cycle Ergometer Protocol) provides essential guidance on prognosis in those with CF who are 10 years of age and older [41]. Engaging in exercise in warm environments should be done with caution as those with CF have a low tolerance to heat stress [42, 43]. Children with CF should be extra vigilant about replacing their fluid loss and electrolytes with exercise because compared to healthy children, patients with CF have higher concentrations of sodium in their sweat [43], lose more fluid, and underestimate their fluid needs [42]. In more severe cases of CF, heart rate and even oxygen saturation should be monitored during exercise sessions to ensure children are exercising within healthy physiological limits [44]. Care should also be taken in gym environments to prevent disease transmission and cross-contamination with other CF patients (wear gloves and clean equipment, don’t exercise in groups with other individuals with CF).

Based on the evidence for pediatric patients with CF, we provide the following exercise suggestions and considerations (summarized in Table 1):

1. **Aerobic cardio-respiratory exercise:** Moderate intensity aerobic exercise (~ 70% of maximum heart rate) has been demonstrated to improve lung function and aerobic capacity [45]. Children with CF should take part in aerobic exercise at minimum two times a week in 30–45 min sessions; but previously sedentary individuals should build up to these sessions in a progressive manner.
2. **Anaerobic type exercise:** Anaerobic activities for children and adolescents often mimic the typical nature of children’s play, and can include running and jumping. Anaerobic sports include volleyball, fencing, track and field, and some swimming events among others. There is some evidence for sustained benefits from anaerobic training when children with CF participate in ~ 30 min sessions of 20–30 s bouts of anaerobic work (maximal or close to maximal effort) with 3 sets of 3–5 repetitions within a set [34]. Children should rest for three times the duration of the exercise (e.g., a 30s bout of exercise would need a 90s rest following the bout), with a longer duration of rest (at least five minutes) in between sets. Aerobic and anaerobic training sessions can be interspersed for recovery and maximal benefit to the patient.
3. **Resistance training:** Resistance training for children and adolescents with CF has been demonstrated to be safe and efficacious [30]. Emphasis should be placed on body-weight exercises (push-ups, lunges, and squats). Any strength training with weights should be done in a supervised environment under the direction of a qualified exercise professional. A moderate intensity workload is 70% of one-repitition maximum (1 RM) for different exercises at 3–5 sets of 10 repetitions [33]; however children should start off with low intensity workloads and build to higher workloads in a progressive manner.
4. **Flexibility and mobility training:** Flexibility and stretching activities should be considered for children with CF. For example, yoga may improve flexibility while conferring both mental and physical benefits (but hot yoga should be avoided due to heat intolerance in CF). A focus on developing the postural muscles, including chest stretching, is highly recommended [46].

ii. Asthma

- *Pathophysiology of Exercise Intolerance in Pediatric Patients with Asthma*

Asthma is defined as a heterogeneous disease characterized by chronic inflammation of the airways [47]. Symptoms such as wheezing, coughing, shortness of breath, chest tightness, and variable expiratory flow are used to establish a diagnosis [47]. The global prevalence of asthma among children is estimated to be 14% [48]. The prevalence varies by sex, such that males have a higher prevalence at a younger age, while females have a higher or similar prevalence post-puberty [49].

Asthma can be allergic or non-allergic in nature, and as such, acute bronchoconstriction can be provoked by a variety of triggers. The increase in ventilation associated with exercise is a trigger in approximately 90% of those with asthma [50]. There are two hypotheses that explain exercise-induced bronchoconstriction (EIBC): the osmotic and the thermal hypothesis. The osmotic hypothesis suggests that an increase in ventilation during exercise leads to an increase in water loss in the airways, triggering EIBC. The thermal hypothesis suggests that the increase in ventilation during exercise leads to cooling of the airways. The subsequent rewarming of the airways following exercise (reactive hyperemia) is thought to trigger EIBC [51]. EIBC likely occurs as a result of both the osmotic and thermal hypothesis.

EIBC can be prevented effectively by using a short-acting bronchodilator 15 min prior to exercise [52]. However, the perceived stigma associated with using medication means that children often do not take their medication prophylactically [53]. There are other ways in which EIBC can be prevented. For example, a high intensity or variable intensity warm-up [54]. Further, controlling for other triggers may reduce the severity of EIBC symptoms. For example, environmental factors such as cold-dry air exacerbate EIBC, thus, exercise can be performed in a warm-humid environment for prevention [55].

Asthma can vary in severity, but it is important to note that asthma control can be achieved for all levels of asthma severity. Unless asthma is poorly controlled, exercise intolerance should not be a limiting factor among children with asthma. Some children with asthma may be less physically active due to fear of EIBC, however, their PA levels do not differ from those of healthy age-matched peers [56]. Of note, children with newly diagnosed asthma may have lower fitness levels and exercise capacity [57], and children with asthma are more likely to be obese [58], which may lead to exercise intolerance (see section 4).

b. *Exercise in Pediatric Patients with Asthma*

Regular aerobic exercise improves asthma symptoms and thus, asthma control levels. Studies have shown that exercise leads to fewer hospital visits, less medication use, less wheezing, less bronchial reactivity, and better quality of life [59–63]. However, it should be noted that regular exercise is not associated with improvements in lung function [64]. In other words, exercise can improve asthma control, but may not impact disease severity.

With regards to aerobic exercise, there are two main considerations for children with asthma; the mode of exercise and the intensity of exercise. The mode of exercise is important as some exercises are conducted in less asthmogenic environments than others. For example, swimming in an indoor pool provides a warm-humid environment i.e. one that is less asthmogenic than running outside on a cold-dry day. Swimming is therefore often recommended to children with asthma.

The intensity of exercise is important as it is directly related to the ventilatory response [65]. Thus, exercise that is performed at a lower intensity or allows for ventilation to recover, might be safer. The latter in particular is referring to HIIT, that is, exercise sessions that include bouts of near maximal exercise with intermittent recovery. Although counterintuitive, this form of exercise is well-tolerated in children with asthma as the brief intervals of high intensity exercise are followed by recovery intervals which allow ventilation to recover [66]. Generally, aerobic exercise is well tolerated in children with asthma, and is not expected to lead to adverse events if medication is available [67].

There is a lack of data on the acute response or chronic adaptations associated with anaerobic exercise, including resistance training, in children with asthma. Of the studies available, it appears that children with asthma have a lower anaerobic capacity than healthy children [68], and that anaerobic exercise induces mild airway obstruction [69]. Further research is needed in this area.

Finally, there is no evidence to suggest that flexibility or mobility exercises are associated with improved asthma control in children. Some studies have shown that yoga may be beneficial for children with asthma; however, these effects are similar to sham yoga or breathing exercises [70]. There is no evidence that the acute response or chronic adaptations that result from flexibility or mobility exercises leads to improved asthma control.

c. *Practical Applications for the use of Exercise as Medicine in Pediatric Patients with Asthma*

There are currently no recommendations for exercise in the National Asthma Education and Prevention Program Guidelines for the Diagnosis and Management of Asthma or the Global Initiative for Asthma Guidelines [71]. Exercise prescription in children with asthma requires guidance on medication use and avoidance of triggers. Specifically, children should be given an asthma action plan that includes information on warming up prior to exercise, use of short-acting bronchodilators prior to exercise, and tips on management of additional triggers such as wearing a face mask if exercising on a cold day outside. If appropriate guidance is provided, children with asthma can follow guidelines for healthy children of similar levels of fitness.

Based on the evidence in pediatric patients with asthma, we provide the following exercise suggestions and considerations (summarized in Table 1):

1. **Aerobic cardio-respiratory exercise:** Children with well-controlled asthma who use their mediation prior to exercise should perform 60 min of MVPA every day as outlined in the PA guidelines [2, 5]. Those who are deconditioned or sedentary or who have suboptimal asthma control, should start with a lower intensity and shorter duration but progressively increase to meet the guidelines. Choosing the mode of aerobic activity is critically important. Those who have a negative and severe response to cold-dry air should avoid exercise outdoors in the winter, or sports such as ice-hockey and ice-skating; those sensitive to smells and chemicals may choose to avoid swimming in a chlorinated pool; and those sensitive to environmental allergens should avoid exercise outdoors, particularly in the spring.
2. **Anaerobic type exercise:** Anaerobic exercise typically induces a significant increase in ventilation and is likely to induce asthma symptoms, unless performed in an intermittent manner similar to HIIT, that is, an exercise protocol that allows ventilation to recover. Due to lack of evidence at this time, it is difficult to say whether anaerobic exercise should be prescribed to children with asthma.
3. **Resistance training:** There is no evidence to suggest that resistance training is unsafe for children with asthma. In fact, among deconditioned children with asthma, resistance training may be a safe way to initiate an exercise program, as low to moderate intensity resistance exercise does not significantly increase ventilation, and therefore is unlikely to induce bronchoconstriction. Further, the physiological adaptations that result from resistance exercise will likely improve tolerance of other daily activities. Recommendations outlined by [72] can be followed. Briefly, a resistance exercise program can begin 2–3 times per week on non-consecutive days. Children should start with 1–2 sets and 8–15 repetitions, and should start with moderate resistance workloads.
4. **Flexibility and mobility training:** Children with asthma can participate in flexibility training such as yoga as it is unlikely to induce respiratory symptoms. It should be noted however, that there is little evidence for disease-specific benefits.

## Congenital heart disease

- *Pathophysiology of Exercise Intolerance in Pediatric Patients with Congenital Heart Disease (CHD)*

Congenital heart disease (CHD) refers to any type of inborn cardiac defect, of which moderate-severe defects are present in 6/1000 live births [73]. Medical advancements have improved the survival rates for patients with CHD. Approximately 90% of children with a repaired CHD defect will survive into adulthood [74]. The cause of exercise intolerance in children with CHD is multifactorial, resulting from external influences causing hypoactivity as well as hemodynamic limitations caused by their heart defect [75].

In complex CHD defects, sinus node dysfunction may affect heart rate responsiveness during exercise stress. Pulmonary and musculoskeletal disorders may also contribute to an impaired exercise response in this patient population [76]. Exercise intolerance may place young CHD patients at an increased risk of developing co-morbidities, including obesity, type 2 diabetes, depression, and anxiety.

b. *Exercise in Pediatric Patients with CHD*

Exercise interventions have demonstrated some improvements in maximal exercise capacity in pediatric patients with CHD. A recent systematic review reported increases in VO_2peak_ averaging 8% in 621 children with CHD participating in regular aerobic exercise training programs [77], with no patients experiencing adverse exercise-related events. However, improvements in VO_2peak_ following aerobic and combined aerobic and resistance exercise interventions are equivocal, with some studies reporting no improvement [78], and others reporting an increase in VO_2peak_ of up to 19% [79]. For example, one study reported a 16% increase in VO_2peak_ following a 12-week exercise intervention (60 min facility-based intervention, 2 x per week, including 45 min of combined aerobic and resistance based activities) in 16 children with CHD [80]. This improvement was sustained 7 months following the program [80].

Data from a systematic review of physical exercise training programs in pediatric patients with CHD found that most studies focused on 12 week training programs, with sessions held 3 times per week and training intensity set at a percentage of the individuals peak heart rate. While systematic review data largely showed a positive change in the main outcome measure after the training period (72% of studies) and no negative findings reported (0/31 studies), the long-term outcomes data (e.g. adherence and health outcomes) are limited and further study is needed [77].

c. *Practical Applications for the use of Exercise as Medicine in Pediatric Patients with CHD*

Exercise interventions are generally safe, feasible, and beneficial in children with CHD [81, 82], with the exception of those patients with heart rhythm disorders [75]. The patient’s cardiologist should be consulted regarding any PA or exercise restrictions prior to program implementation. CHD patients on anticoagulant therapy and with implanted devices (e.g. pacemakers) should avoid contact sports; exercise in a thermoneutral environment is also encouraged to prevent heat-related illness and negative cardiac responses [75, 82].

Regular clinical assessment of maximal exercise capacity in patients with CHD may be useful to monitor disease progression and evaluate safety guidelines for participation [83]. A maximal cardiopulmonary exercise test may have prognostic value, but may also be used to determine if any impairment in peak exercise performance exists or if an abnormal heart rhythm develops during exercise stress. Holter monitoring can be performed to examine any heart rhythm abnormalities over a 24 h or 48 h period. These clinical tests can be used to provide exercise clearance for children and adolescents with CHD.

Based on the evidence in pediatric patients with CHD, we provide the following exercise suggestions and considerations (summarized in Table 1):

1. **Aerobic cardio-respiratory exercise:** A recent consensus statement from the European Pediatric Cardiology Association stated that most children with CHD should participate in 60 min per day of MVPA (40–85% of VO_2peak_), which matches the current PA recommendations for otherwise healthy children [82]. Progression in exercise duration (e.g. shorter exercise bouts of PA, while slowly and consistently working towards 60 min of endurance type exercise) is recommended [2]. Children with some specific CHD defects, including Tetralogy of Fallot and Functional Single Ventricle patients (e.g., Fontan patients) are recommended to limit their aerobic exercise to low to moderate intensity (rather than MVPA) [82].
2. **Anaerobic type exercise:** No studies have examined the safety and efficacy of HIIT or anaerobic training in pediatric patients with CHD. Therefore, the safety and effectiveness of higher-intensity exercises have not been determined in CHD cohorts, and high intensity interval training should be avoided until further evidence is reported.
3. **Resistance training:** Low-to-moderate intensity strength training of individual muscle groups is safe for the majority of CHD patients (i.e., a high number of repetitions 10-15, with lower resistance) [82, 84]. High intensity strength training has not been examined in this cohort, and may increase the risk of injury and could increase blood pressure, decrease cardiac output, and cause bradycardia in some patients with CHD [85]. High intensity strength training should be avoided in this group until further research is available.
4. **Flexibility and mobility training:** Dynamic stretching exercises have been included as a component of numerous exercise intervention studies (such as warm-up prior to aerobic or resistance training) [80], therefore children with CHD can likely safely participate in flexibility training. However, there is little evidence for disease-specific benefits of flexibily training.

## Metabolic disease

i. Obesity & Type 2 Diabetes

- *Pathophysiology of Exercise Intolerance in Pediatric Patients with Obesity & Type 2 Diabetes*

We are currently experiencing a worldwide epidemic of obesity, with pediatric obesity levels on the rise [86–93]. Obesity is defined as an excessive nonessential adipose tissue accumulation [94], with body mass index (BMI) percentiles commonly used to classify obesity status in children (overweight: 85th to 95th percentile; obese: 95th to 99th percentile; severely obese ≥99th percentile) [95]. There are many complictions associated with obesity, one of which is type 2 diabetes [96–98]. Type 2 diabetes is a chronic metabolic disorder marked by hyperglycemia and results from an inadequate response to insulin [94]. In one study of children and adolescents in the United States, the overall unadjusted incidence rates of type 2 diabetes increased by 7.1% annually (from 9.0 cases per 100,000 youths per year in 2002–2003 to 12.5 cases per 100,000 youths per year in 2011–2012) [99].

Several pathophysiological adaptations that may affect exercise tolerance emerge in the cardiac, respiratory, endocrine, and musculoskeletal systems as a result of obesity. Cardiac pathophysiologic adaptations may include increases in cardiac output, blood pressure, and cardiac hypertrophy. Respiratory pathophysiologic adaptations may also occur and include an increased ventilation frequency, and decreased respiratory muscle efficiency [100], which may result in a greater metabolic demand. Musculoskeletal pathophysiologic adaptations may include intramyocellular fat accumulation, impaired phosphorus energy metabolism, and increased stress and pain in weight-bearing, lower-body joints [101].

Obesity often precedes type 2 diabetes by increasing the production of free fatty acids which interfere with insulin receptor signaling and glucose transport. Lipid accumulation can occur within skeletal muscle (i.e., intramyocellular lipid deposition) and impair both insulin signaling [102] and mitochondrial function [103]. Furthermore, pathophysiological processes of type 2 diabetes directly contribute to exercise intolerance, which has been described in detail in a recent review of the literature [104]. Chronically low levels of PA in obese children and adolescents [105, 106] with or without type 2 diabetes, may further contribute to pathophysiological deconditioning and exercise intolerance.

b. *Exercise in Pediatric Patients with Obesity and Type 2 Diabetes*

Exercise interventions represent an important clinical strategy for the prevention of obesity and co-morbidities in adolescents [107, 108]. In particular, aerobic exercise interventions have been shown to significantly decrease adiposity [109–112], improve cardiometabolic risk [113, 114], increase muscle mass [115, 116], and improve cardiorespiratory function [117, 118] in obese adolescents. Anaerobic activities are also not restricted for children with obesity (for example, during play). A recent study determined that HIIT (12 intervals at 120% of maximal aerobic running speed, 6 min in total) is more effective at reducing skinfold thickness than low intensity interval training (16 intervals at 100% of maximal aerobic running speed, 8 min in total) [119]. In comparison, a recent systematic review and meta-analysis of 40 studies reported that resistance exercise has minimal effects on body composition, but moderate to large effects on muscular strength [120].

Pediatric patients with type 2 diabetes reportedly perform as much as 60% less MVPA than peers without diabetes [121]. Nassis and colleagues explored the effectiveness of a 12-week aerobic exercise training program (40 min of aerobic exercise performed 3 times weekly) on insulin concentration (via 2 h oral glucose tolerance test) in overweight adolescent girls [122]. They reported a decline in insulin concentration independent of changes in body mass, suggesting that moderate levels of aerobic exercise may have a positive effect on insulin sensitivity. Likewise, in a study of 22 adolescent males, a 45% increase in insulin sensitivity was observed following a 16-week resistance training program (1 h of resistance training performed 2 x per week) [123]. Therefore, moderate levels of exercise (2 h per week), independent of exercise modality, may be associated with significant improvements in insulin sensitivity and resistance in youth with Type 2 Diabetes [124].

In a recent systematic review meta-analysis conducted to compare aerobic, resistance, and combined exercise training on insulin resistance in obese adolescents, aerobic exercise training was associated with the most favourable changes in fasting insulin levels and insulin resistance marker (HOMA) when compared to other training modalities [125]. These findings suggest that exercise interventions, even moderate levels of aerobic or resistance exercise, may be effective for improving peak exercise capacity and/or regulating glucose metabolism in obese children/adolescents with or without type 2 diabetes.

c. *Practical Applications for the use of Exercise as Medicine in Pediatric Patients with Obesity and Type 2 Diabetes*

Pediatric patients with obesity can accumulate PA amounts in shorter exercise bouts throughout each day with the focus on rate of perceived exertion and target heart rate (as opposed to performance based outcomes like speed), particularly if the child is previously sedentary and physiologically deconditioned. PA progressions should be implemented as physiological adaptations are noted [6]. Modifications can be considered where obesity-related pathophysiological adaptations impair cardiopulmonary function during intensive exercise. A prolonged warm-up may be recommended to allow the obese child to reach a comfortable steady-state [126]. Non-weight bearing activities such as cycling or swimming may be advised for obese children due to the increased risk of osteoarthritis in weight bearing joints [127].

For children with type 2 diabetes, The American Academy of Pediatrics has recommended an integrated clinical treatment approach, emphasizing a combination of medication (such as Metformin) as well as diet and PA modifications to achieve glucose control [128]. Most pediatric patients with type 2 diabetes should perform at least 60 min of daily PA [2]. Older adolescents or those with greater exercise intolerance may not be able to safely perform this amount of PA initially, however, as described above they should work on accumulating short bouts of PA on a daily basis. In fact, exercise training involving aerobic intervals and/or resistance training may actually enable individuals with low cardiorespiratory fitness to achieve a moderate level of PA. Care should be given to ensure that children with type 2 diabetes and carefully monitor their blood sugar before and after exercise bouts, with the aim to maintain a controlled blood sugar status.

Based on the evidence in pediatric patients with obesity and/or type 2 diabetes, we provide the following exercise suggestions and considerations (summarized in Table 1):

1. **Aerobic cardio-respiratory exercise:** Pediatric patients with obesity and type 2 diabetes should be encouraged to engage in a prolonged warm-up and cool-down for injury prevention, and to progressively increase exercise duration (e.g. working toward 60 min of moderate-to-vigorous aerobic exercise per day) [2]. Lower impact or non-weight bearing, moderate-intensity aerobic activities may be advised for orthopedic injury prevention and exercise in thermoneutral environments is encouraged if children have compromised ability to dissipate heat in children who are obese.
2. **Anaerobic type exercise:** Interval type exercise may be feasible for obese youth to enable higher work rates during shorter bursts of activity [129–131]. There is some evidence to suggest that children with obesity can perform HIIT exercise 2 times per week, at 70–85% of their max HR during work bouts [119]. This protocol may be used to inform individualized exercise prescriptions, however few studies to date have used HIIT training in obese youth, and therefore optimized prescription suggestions are lacking. Due to lack of evidence at this time, it is difficult to say whether anaerobic exercise should be suggested to children with type 2 diabetes.
3. **Resistance training:** Regular strength training of large muscle groups (3 days per week) is recommended to promote muscle strength and insulin sensitivity [132, 133]. Resistance-based exercise may also be beneficial prior to initiating an aerobic exercise program as it may help to build muscle strength and exercise capacity. Training can be completed in 1 to 3 sets of up to 15 repetitions, 2–3 days per week. Increases in load may occur following the successful completion of 15 repetitions in good form.
4. **Flexibility and mobility training:** Children with obesity and/or type 2 diabetes can likely safely participate in flexibility training [2]. Although there are no EX-RCTs that focus on flexibility training, one study in obese youth incorporated yoga-based breathing in their multi-component exercise program [134]. Research is still needed to determine the association between stretching activities and weight loss/ insulin sensitivity in children.

## Systemic inflammatory/autoimmune disease

i. Juvenile Idiopathic Arthritis (JIA)

- *Pathophysiology of Exercise Intolerance in Pediatric Patients with JIA*

Juvenile Idiopathic Arthritis (JIA) is a common chronic disease that presents during childhood; in fact, JIA affects one in every 1000 children and teenagers in Canada [135, 136], and close to 300,000 children in the United States [137]. JIA is an autoimmune disease that results in joint-specific inflammation that can lead to damage of bone and cartilage [135].

Children with JIA have poor PA levels, reduced fitness, and decreased exercise tolerance [138, 139]. As well, poor anaerobic fitness is strongly associated with reduced functional ability in JIA [140]. There are many mechanisms that contribute to exercise intolerance in this cohort. For example, inflammation and joint degradation can result in pain and difficulty with moving. Muscle wasting and weakness is a common symptom that directly results from JIA, which may contribute to difficulty in maintaining PA levels [139]. A vicious cycle of inactivity including: joint pain and muscle weakness lead to reduced PA levels, may contribute to muscle atrophy, pain, and deconditioning. Moreover, poor exercise habits may also contribute towards increased body weight, or obesity, resulting in increased joint loads, and exacerbate pain resulting in further reductions in activity participation [141].

b. *Exercise in Pediatric Patients with JIA*

The use of exercise as medicine in children with JIA has been investigated in some non-randomized and EX-RCTs. In 2008, Tim Takken and co-workers published a Cochrane review on exercise therapy in JIA [142]. They identified three eligible EX-RCTs representing 212 children with JIA, that employed an exercise therapy protocol [143]. Pooled outcome measures included functional ability, quality of life, and aerobic fitness. These authors reported that all outcome measures improved with exercise; while these improvements were clinically meaningful, the improvements did not reach statistical significance in their analyses [142]. However, the evidence is limited by the low number of EX-RCTs, as well as the large variety in the type of exercise prescribed and outcomes assessed in the published trials. Perhaps most importantly, none of the exercise interventions evaluated in the review reported adverse events; therefore, exercise appears to be safe in children with JIA [142].

Since the Cochrane review article, new data has emerged that provides further, support for the effectiveness of exercise in JIA. An EX-RCT of 48 children ages 8–13 years old, who participated in a 14-week cognitive behavioral intervention to increase PA levels, reported improvements in self-reported and objective PA measures as well as improvements in exercise capacity within the intervention group that persisted to three months post intervention [143]. The control group experienced a decline in exercise capacity over the same time period, however the differences between the experimental and control groups were not statistically significant [143]. School absences also decreased and physical education participation increased significantly in the exercise group, while school absences increased in the control condition [143]. A recent non-randomized controlled exercise intervention reported similar findings. In this study, 30 patients with JIA and 20 age matched healthy controls were prescribed aerobic walking (4 days per week) and active and passive range of motion exercises for 8 weeks. The exercise program significantly improved physical parameters in children with JIA including VO_2peak_ which changed from 32.5 ± 6.6 ml/kg/min at baseline to 35.3 ± 7.9 ml/kg/min post intervention [138].

Preliminary evidence regarding the safety and efficacy of resistance training programs suggests positive effects in children with JIA. For example, a study of 7 participants with JIA taking part in a home-based resistance training program, 3 days per week, for 6 weeks of training reported no adverse events and no exacerbations in pain. Following the exercise program, there was a significant increase in vastus lateralis thickness from pre- to post-training as assessed by ultrasound. These results support the safety and feasibility of resistance training in children with JIA [144]. Significant improvements were also reported in physical function and quality life following a EX-RCT of 43 children taking part in a 12 week strengthening and stretching program [145].

c. *Practical Applications for the use of Exercise as Medicine in Pediatric Patients with JIA*

The data indicates that exercise programs, both aerobic and strength training, are safe and feasible in children with JIA. The evidence for the efficacy of these programs is limited, but currently supports small positive effects of PA participation on exercise capacity, muscle strength, school attendance and participation in physical education in this cohort.

Ideally, children with JIA should be followed by a multidisciplinary team involving primary care physicians, as well as physiotherapists and qualified exercise professionals, when returning to activities, especially high loading sports, as children with JIA may have changes to neuromuscular function that increase their chance of injury [146]. Furthermore, there are multiple subtypes and severities of JIA, some of which can result in organ inflammation including the heart. Therefore, children should be cleared by a physician to ensure that there are no cardiovascular complications with exercise (and if there are, make appropriate exercise accommodations).

There are several contraindications to participation in PA in children with JIA that have been noted in the literature and should be considered. Children with JIA should not participate in physical activities when they are febrile, exercise should be conducted within pain limits, and should include activities that do not exacerbate joint pain [141, 147]. Following a flare-up of JIA, reintroduction to exercise should be progressive and gradual [141]. Children with moderate to severe knee or hip pain should not participate in high impact or vigorous physical activities [148]. Contact sports in children with severe bone loss or spinal arthritis should be avoided [141, 147] due to fracture or spinal cord injury risk. Although exercise has been demonstrated to improve bone health in JIA [149], it is also important to approach impact/weight bearing exercise in a progressive nature to avoid micro- or larger fractures. As well, appropriate eye protection and dental protection should be considered for those with uveitis or temperomandibular joint disease in contact sports [141].

Based on the evidence in pediatric patients with JIA, we provide the following exercise suggestions and considerations (summarized in Table 1):

1. **Aerobic cardiorespiratory exercise:** Encourage prolonged warm-up and cool-down for injury prevention, and the use of progression in exercise duration [44, 138]. Lower impact, moderate-intensity aerobic activities (for example, swimming) may be advised for injury prevention and comfort following a disease flare-up or in those with JIA. However, since weight-bearing activity is beneficial to bone health, this type of exercise should be encouraged, progressively. Exercise may be safely performed between 65 to 85% of a child’s calculated maximum heart rate and should be performed two to three days per week [142, 150], in a progressive manner, up to 45–60 min in duration. Similar considerations as the CHD group should be adhered to if children have cardiovascular complications.
2. **Anaerobic type exercise:** The safety and effectiveness of HIIT has not been determined in the JIA cohort. Since joint health, fracture risk, and inflammatory response are concerns in JIA, we would recommend avoiding HIIT in children with JIA until further research is conducted.
3. **Resistance training:** Children with JIA may take part in a similar resistance program as healthy children, but special attention should be paid to performing the exercises in a range of motion that does not exacerbate pain [151]. The published literature suggests that low-to-moderate intensity strength training is likely safe for the majority of patients with JIA (including a high number of repetitions with lower resistance). Initial intensity should be set at 60% of estimated one repetition maximum and be slowly progressed to 75% [151]. Training can be completed in 1 to 3 sets of up to 15 repetitions, 2–3 days per week. Increases in load may occur following the successful completion of 15 repetitions in good form [151]. If using progressively heavier weights, exercise supervision is recommended. Slow, controlled, active resistance exercises with the use of therapy bands is also a recommended type of exercise for this cohort [144, 145].
4. **Flexibility and mobility training:** Children with JIA may participate in flexibility training such as yoga without restrictions to increase range of motion. Yoga, Qigong or tai chi may be beneficial for this cohort to encourage joint movement and prevent stiffness [150].

## Cancer

- *Pathophysiology of Exercise Intolerance in Pediatric Patients with Cancer*

Pediatric cancer is the number one cause of disease-related mortality in individuals under the age of 20 [152], and nearly 1000 new cases of childhood cancer are diagnosed annually in Canada alone [153]. As treatments have become increasingly effective, 5-year survival rate has improved to ~ 85% [154], however, significant morbidity is experienced during survivorship as a consequence of disease treatment [155–157].

The disease course is characterized by aberrant cell growth and division causing dysfunction of tissues and organ systems as dysfunctional cancerous cells replace healthy, functional cells [158]. Treatment includes surgical resection of the tumour, local or full body radiotherapy, chemotherapy, or a combination of these treatments. Chemotherapy and radiotherapy are both nonspecific, cytotoxic treatments and are largely responsible for long-term health and functional consequences of pediatric cancer, including exercise intolerance [156, 157, 159].

Exercise intolerance in pediatric cancer patients manifests impaired aerobic and anaerobic fitness, lower muscular strength, and impaired neuromuscular coordination, balance, and flexibility [155, 160–163]. Indirect consequences of treatment, such as acute side effects - nausea, extreme fatigue, anemia, immunosuppression- and duration of hospitalization also impact a patient’s ability and willingness to be physically active during treatment [160, 164]. Lasting impairments to exercise tolerance are a direct physiological consequence of treatment, varying based on type of treatment and dose [157, 159, 165]. For example, intravenous chemotherapy and total body irradiation cause systemic inflammation and oxidative stress, which may damage vascular endothelial cells and skeletal muscle cells [156, 166, 167], impairing oxygen delivery and aerobic metabolism in skeletal muscles during activity [156, 167]. Anthracycline chemotherapies can cause lasting damage to cardiomyocytes, further limiting aerobic function via central mechanisms [168, 169].

b. *Exercise in Pediatric Patients with Cancer*

Exercise interventions reliably demonstrate improved aerobic fitness and muscle strength in pediatric cancer patients during treatment and off treatment during early survivorship [161, 170, 171]. Exercise programs vary in exercise type, duration, patient population, and treatment phase, resulting in varied reports of changes in fitness outcomes. A 12-month home-based nutrition and fitness intervention for leukemia patients aged 4–10 years old, was tested against usual care and resulted in increased self-reported PA levels and cardiovascular fitness at 6 months and 12 months [172]. While this program was successful, other home-based programs have shown small or null effects [173, 174]. Conversely, hospital-based programs are reliably successful at achieving fitness gains in participants. For example, a 12-week aerobic intervention performed for 30 min 3 times per week under the supervision of a physiotherapist or a parent resulted in increased aerobic fitness during induction phase of chemotherapy for acute lymphoblastic leukemia [175].

Many studies use a mixed exercise intervention, with positive results for both aerobic and strength training [176, 177]. In one mixed resistance and aerobic training program for stem-cell transplant recipients, exercise training resulted in increased VO_2peak_ by ~ 5.2 ml/kg/min and increased 1 repetition max for bench press, leg press, and seated row [176]. Exercise interventions appear to be feasible in patients during all points of treatment. Importantly, although improvements have been found following exercise interventions, patients still remain below the fitness level of their peers, and findings related to continued participation and fitness improvements are variable [170].

c. *Practical Applications for the use of Exercise as Medicine in Pediatric Patients with Cancer*

Due to the variable degree of exercise intolerance in pediatric cancer patients, an individualized assessment of general fitness abilities should be conducted by an exercise professional who has knowledge of expected side-effects of their treatment regimen. For patients on treatment who may be immunosuppressed, care should be taken to ensure all equipment is sterilized to avoid risk of infection. Children with platelet counts below 10,000 per μL should not exercise [161]. Overall, pediatric cancer patients should strive to achieve national PA guidelines [2], however this may need to be done in smaller and more frequent doses to accommodate the acute side-effects of treatment. Fatigue is a common side-effect of treatment, and this should be taken into consideration. If possible, programs should be supervised by an exercise professional, as home-based programs are less reliably efficacious.

Based on the evidence in pediatric patients with cancer we provide the following exercise suggestions and considerations (summarized in Table 1):

1. **Aerobic cardio-respiratory exercise:** Aerobic training programs can be started at the beginning of treatment between 2 and 4 days per week progressing to 3–5 days per week. Exercise intensity can begin ~ 40% max HR working up to 70%, for patients on treatment, or higher for patients off treatment with no concerns of immunological complications. Interval-type formatting may also be appropriate for transitioning towards higher intensities. Duration should be 30–45 min but may be broken into shorter blocks of time to meet this according to patient’s side-effects and fatigue level. Specific recommendations for children with apparent cardiomyopathy from anthracycline treatment can be found in a more detailed review [168].
2. **Anaerobic type exercise:** Due to lack of evidence at this time, it is difficult to say whether anaerobic exercise should be prescribed to children with cancer.
3. **Resistance Training:** Resistance training should be included in a patient’s exercise plan 1–3 days per week on non-consecutive days and multiple recovery days between workouts should be given if immunosuppression is a concern. Higher reps (12-15) with lower resistance (60% 1 RM) should be used initially, progressing towards higher resistance (75% 1 RM) with fewer reps (10-12) as strength is improved. Body weight and resistance bands are appropriate for younger patients. Resistance exercises should be provided by a trained exercise professional and exercise should be supervised to ensure proper technique is used.
4. **Flexibility and mobility training:** Pediatric cancer patients may also benefit from stretching and balance programs, such as yoga. Activities emphasizing neuromuscular coordination and functional movement skills may help minimize physical literacy deficits associated with inactivity. However, there is little evidence for disease-specific benefits.

## Discussion

It is evident that children with the chornic diseases reviewed herein can benefit from PA and exercise interventions for the same reasons as healthy children, in addition to disease-specific benefits. Overall, exercise appears to be a safe and efficacious intervention across the chronic diseases reviewed, as long as disease specific and individual needs are carefully considered. It is important to note that the current review is a narrative review of the literature, not a systematic review. As such, there is greater potential for bias, as the quality of published data was not assessed. Thus, our practical applications for exercise should be used as a starting point in determining the use of exercise as medicine in pediatric chronic disease rather than as specific guidelines. While a position statement (endorsed by The Canadian Paediatric Society and the Canadian Academy of Sport Medicine) was created for some pediatric chronic diseases [141], there is still a lack of high quality data to make recommendations at this time. This narrative review is a necessary first step towards filling a gap in the literature in the area of exercise as medicine in pediatric chronic disease and is critical, since producing high quality data and evidence-informed recommendations may take several years.

We examined five common pediatric chronic disease areas in this paper including respiratory, congentical heart, metabolic disease, systemic inflammatory/autoimmune, and cancer. We chose to include these categories of chronic diseases because they are prevalent pediatric diseases [9], however our list of included diseases is not exhaustive and there are many other chornic diseases that were not included in the current review. We focused our manuscript on the benefits of PA on physiological outcomes within the context of chronic disease; we did not evaluate the effects of PA on psychological or social outcomes. These are important future considerations as well.

## Conclusions

Children with chronic disease present with specific pathophysiologies that each uniquely contribute to exercise tolerance. International PA guidelines suggest that children should accumulate 60 min of daily MVPA; but these guidelines do not consider disease-specific factors that contribute to exercise intolerance in chronic diseases, and are non-specific. Comprehensive chronic disease PA guidelines are necessary, as they consider the safety of exercise, the dose, frequency, intensity, time and type of exercise on a per-disease basis. PA suggestions, such as the ones presented in this manuscript, provide a staring point that may help inform clinicians and health care teams working with pediatric chronic disease cohorts on how to appropriately use *exercise as medicine*. Based on literature reviewed, it appears that regular exercise is beneficial for children with CF, asthma, CHD, obesity, type 2 diabetes, JIA, and cancer. We provide our practical applications of PA with the understanding that high quality, EX-RCTs are still needed in many disease areas, to help determine the best exercise program to provide the most effective and safe improvements. There remains a large gap in the literature regarding PA/exercise suggestions and recommendations in pediatric chronic disease. This area of research should be a continued priority globally as PA/exercise is a powerful tool to improve health.

## Abbreviations

BMI: Body mass index
CF: Cystic Fibrosis
EIBC: exercise-induced bronchoconstriction
EX-RCTs: Randomized controlled exercise intervention trials
HIIT: High intensity interval training
JIA: Juvenille Idiopathic Arthritis
MVPA: Moderate to vigorous physical acitivity
PA: Physical activity
1 RM: 1 repitition maximum
VO_2peak_: Maximal aerobic capacity
